# A significant association between examination results and self-satisfaction with English language proficiency: preliminary findings among pre-clinical undergraduates

**DOI:** 10.1186/s13104-018-3912-6

**Published:** 2018-11-12

**Authors:** Madushika Wijesundara, Chamindi Wijerathna, Kasun Wijerathna, Rasangi Wijerathna, Srimali Wijethunga, Ashan Wijewardana, Anuprabha Wickramasinghe, Devarajan Rathish

**Affiliations:** 1 Faculty of Medicine and Allied Sciences, Rajarata University of Sri Lanka, Saliyapura, Sri Lanka; 2 Department of Psychiatry, Faculty of Medicine and Allied Sciences, Rajarata University of Sri Lanka, Saliyapura, Sri Lanka; 3 Department of Pharmacology, Faculty of Medicine and Allied Sciences, Rajarata University of Sri Lanka, Saliyapura, Sri Lanka

**Keywords:** Medical education, Undergraduates, English proficiency, Peer-revision notes

## Abstract

**Objectives:**

Learning methods and other related factors influence the success of medical undergraduates. This study aims at finding factors associated with the end of pre-clinical stream examination results among medical undergraduates of the Rajarata University of Sri Lanka. The results of this study will inform the tutors to plan and implement teaching methods as well as to guide the social welfare of the undergraduates. In general, we believe this study has the potential to improve the medical undergraduate’s academic performance.

**Results:**

Eighty-six per cent (112/130) of medical undergraduates have passed the examination and rest was referred. Logistic regression revealed a significant association between examination results and self-satisfaction for English language proficiency (P = 0.048). Passing the examination was more likely with high self-satisfaction for English language proficiency [odds ratio = 6.063 (95% CI 1.014 to 36.249)]. Also, a significant association between obtaining a class at the examination and using peer-revision notes (P = 0.019) was revealed. Obtaining a class at the examination was less likely with the frequent use of peer-revision notes [odds ratio = 0.228 (95% CI 0.066 to 0.790)].

**Electronic supplementary material:**

The online version of this article (10.1186/s13104-018-3912-6) contains supplementary material, which is available to authorized users.

## Introduction

Undergraduates entering medical schools are selected through preceding higher academic performances. Although previous academic performance is a suitable prognosticator of achievements at the medical school, it is not a perfect predictor [1]. Reflective and active learning styles were found to be more common among preclinical and clinical undergraduates [2]. Career concerns and goal orientation were related to higher academic performances [3]. Nevertheless, completion of the medical course is not without the involvement of stress [4–6]. However, self-directed learning has been positively associated with knowledge acquisition [7].

Various methods are used by medical undergraduates to prepare for examinations [2, 8, 9]. Reproducing and meaning orientation has its influence on the success of medical undergraduates [10]. Learning styles vary and are subjective. Also, these may be associated with individual comprehension and predict success at examinations [11]. It is a complex task to figure out the study method that best suits an individual. Tutors should be able to cater knowledge according to different learning styles [2]. Most undergraduates use deep and strategic learning styles, while the latter leads to higher examination results [11]. Medical undergraduates prefer problem-based learning than traditional lectures to enhance their learning outcomes [12]. Undergraduates have acknowledged team-based learning and learning from peers [13]. Preclinical academic achievements is a predictor of better performance during the later years at medical school [14]. Therefore, good learning methods introduced during the pre-clinical stage could enhance medical undergraduate’s future performance.

There are factors other than learning methods that can affect the undergraduate’s ability to be an effective learner [15–17]. Positive and negative emotions are paramount in successful learning [18]. Some undergraduates undergo severe stress in completing the course [4, 15, 19]. Majority experience stress related to their academic work and examinations [20, 21]. Stress is highly recorded, especially during the first year [22]. It is a challenging job for them to navigate through the initial years at the medical school. Adequate sleep, less time on social media, and weekend academic work has shown to achieve higher academic performance [16]. Lack of awareness on learning methods, poor physical fitness, inadequate extracurricular activities, psycho-social issues, lack of sleep and unhealthy food practices are some contributive factors for stress and the subsequent academic failure [23, 24]. Change in the medium of learning and living in hostels were also associated with academic related stress [25].

This study was aimed at finding the factors associated with the end of pre-clinical stream examination results of the medical undergraduates of the Rajarata University of Sri Lanka. An important insight is expected which will inform the future generation of undergraduates in effective learning.

## Main text

### Methods

A descriptive cross-sectional study was conducted among the pre-clinical stream medical undergraduates of the Faculty of Medicine and Allied Sciences, Rajarata University of Sri Lanka. This latest medical school of Sri Lanka was established in 2006. It is situated at Anuradhapura, a rural district of Sri Lanka [26], 210 km away from the capital city of Colombo. Medical education at this faculty is conducted in English medium. Around 900–1000 undergraduates are on the roll at a given time and are divided into pre-clinical, para-clinical and clinical streams. The pre-clinical stream includes the first and second year teaching of anatomy, biochemistry and physiology. Lectures, practical sessions, tutorials and dissections are methods used for teaching and learning activities. The end of pre-clinical stream examination is a bar examination to enter the para-clinical stream, and a maximum of four attempts are allowed. It includes theory (multiple choice and short essay questions) and practical examinations. All undergraduates who received their end of pre-clinical stream main examination results in the year 2016 were approached soon after the release of the results and 142 out of 182 (78%) participated in the study.

A self-administered questionnaire was used to obtain data for examination results (outcome variable) and the following independent variables: (1) revision of lecture handouts before the beginning of the study leave, (2) using textbooks, peer-revision notes and online resources, (3) having an effective study group, (4) self-practice of past paper questions, (5) discussing past paper questions in a study group, (6) sense of interference to academic work due to stress and psycho-social issues, (7) duration of uninterrupted sleep and (8) self-satisfaction with time management, English language proficiency, physical fitness, leisure activity, family support, financial support and accommodation. Lecture hand-outs are usually of the whole lecture which includes the detailed subject matter and the list of resources which the undergraduates should refer. Response categories for each of the variables are shown in Tables 1 and 2. The questionnaire was pre-tested among five undergraduates to improve its content, language and sequence.

**Table 1:** Examination results against the variables of interest (passing the examination)

Item	Description	Passed	Referred	Logistic regression		
				P value	Coefficient	Odds ratio (95% CI)
1. Revising lecture notes	Before study leave	100	12	0.177	1.307	3.694 (0.555–24.587)
	During study leave	12	6			
2. Using textbooks	Frequently	106	14	0.443	0.951	2.587 (0.228–29.396)
	Occasionally	6	4			
3. Using peer-revision notes	Frequently	94	15	0.754	0.280	1.323 (0.230–7.606)
	Occasionally	18	3			
4. Using online resources	Frequently	56	10	0.328	− 0.710	0.492 (0.119–2.039)
	Occasionally	56	8			
5. Effective study group	Had	24	3	0.513	− 0.585	0.557 (0.096–3.219)
	Not had	88	15			
6. Self-practice of past multiple-choice questions	Frequently	98	14	0.391	− 1.035	0.355 (0.033–3.779)
	Occasionally	14	4			
7. Self-practice of past short essay questions	Frequently	98	12	0.081	1.756	5.787 (0.806–41.524)
	Occasionally	14	6			
8. Discussing past paper questions in a group	Frequently	70	12	0.455	− 0.605	0.546 (0.112–2.670)
	Occasionally	42	6			
9. Stress related interference to academic work	Minimal	27	4	0.106	1.539	4.661 (0.720–30.159)
	Substantial	85	14			
10. Psycho-social issues related interference to academic work	Minimal	60	14	0.545	− 0.472	0.623 (0.135–2.882)
	Substantial	52	4			
11. Uninterrupted sleep	≥ 8 h	14	3	0.170	− 1.573	0.208 (0.022–1.960)
	< 8 h	98	15			
12. Satisfaction with time management skills	High	61	04	0.081	− 1.392	0.249 (0.052–1.188)
	Less	51	14			
13. Satisfaction with English language proficiency	*High*	*101*	*12*	*0.048*	*1.802*	*6.063 (1.014–36.249)*
	Less	11	06			
14. Satisfaction with physical fitness	High	84	12	0.798	0.212	1.236 (0.243–6.289)
	Less	28	6			
15. Satisfaction with leisure activities	High	55	8	0.203	0.916	2.499 (0.610–10.247)
	Less	57	10			
16. Satisfaction with family support	High	104	18	0.999	− 21.255	0.000
	Less	8	0			
17. Satisfaction with financial support	High	102	16	0.257	− 1.623	0.197 (0.012–3.256)
	Less	10	2			
18. Satisfaction with accommodation	High	72	15	0.081	− 1.632	0.195 (0.031–1.221)
	Less	40	3			

Italic values indicate significance with a p-value of < 0.05

**Table 2:** Examination results against the variables of interest (obtaining a class at the examination)

Item	Description	Passed without a class	Passed with a class	Logistic regression		
				P value	Coefficient	Odds ratio (95% CI)
1. Revising lecture notes	Before study leave	75	25	0.249	− 1.041	0.353 (0.060–2.073)
	During study leave	08	04			
2. Using textbooks	Frequently	78	28	0.561	− 1.031	0.357 (0.011–11.496)
	Occasionally	05	01			
3. Using peer-revision notes	*Frequently*	*73*	*21*	*0.020*	*− 1.480*	*0.228 (0.066–0.790)*
	Occasionally	10	08			
4. Using online resources	Frequently	43	13	0.827	− 0.118	0.889 (0.308–2.564)
	Occasionally	40	16			
5. Effective study group	Had	18	06	0.958	− 0.035	0.965 (0.259–3.602)
	Not had	65	23			
6. Self-practice of past multiple-choice questions	Frequently	70	28	0.190	2.143	8.523 (0.346–209.809)
	Occasionally	13	01			
7. Self-practice of past short essay questions	Frequently	71	27	0.945	0.072	1.074 (0.141–8.168)
	Occasionally	12	02			
8. Discussing past paper questions in a group	Frequently	51	19	0.888	0.079	1.082 (0.363–3.223)
	Occasionally	32	10			
9. Stress related interference to academic work	Minimal	20	07	0.455	− 0.507	0.603 (0.160–2.274)
	Substantial	63	22			
10. Psycho-social issues related interference to academic work	Minimal	42	18	0.252	0.673	1.961 (0.619–6.209)
	Substantial	41	11			
11. Uninterrupted sleep	≥ 8 h	09	05	0.072	1.423	4.148 (0.878–19.595)
	< 8 h	74	24			
12. Satisfaction with time management skills	High	39	12	0.397	− 0.490	0.612 (0.197–1.903)
	Less	44	17			
13. Satisfaction with English language proficiency	High	74	27	0.522	0.640	1.896 (0.267–13.454)
	Less	09	02			
14. Satisfaction with physical fitness	High	60	24	0.820	0.142	1.153 (0.339–3.916)
	Less	23	05			
15. Satisfaction with leisure activities	High	38	17	0.405	0.457	1.580 (0.538–4.639)
	Less	45	12			
16. Satisfaction with family support	High	76	28	0.301	1.416	4.121 (0.281–60.468)
	Less	07	01			
17. Satisfaction with financial support	High	74	28	0.576	0.717	2.049 (0.166–25.340)
	Less	09	01			
18. Satisfaction with accommodation	High	51	21	0.635	0.298	1.348 (0.393–4.624)
	Less	32	08			

Italic values indicate significance with a p-value of < 0.05

Explaining the study, obtaining verbal informed consent and data collection were done by the first six authors. All necessary measures were taken to preserve participant’s privacy and confidentiality. Permanent lecturers at the faculty avoided obtaining consent or collection of the questionnaires to avoid coercion. Data were entered into a Microsoft Excel sheet for analysis (Additional file 1). Descriptive statistics were used to describe data. Undergraduates were grouped into two: those who passed and those who referred at the examination. Also, those who passed the examination were grouped into two: passed without a class and passed with a class at the examination. Logistic regression was performed to determine the significant association between variables of interest and the examination results (P < 0.05). Odds ratios with 95% confidence interval were presented for each variable of interest.

### Results

Out of the 142 undergraduates who participated in the study, 12 were omitted from analysis due to missing data. Eighty-six per cent (112/130) have passed the examination and rest was referred. Out of those who passed, 26% (29/112) had a class.

Eighty-nine per cent (101/113) of undergraduates who had high self-satisfaction for English language proficiency passed the exam, in comparison to 65% (11/17) of those who thought to have less self-satisfaction for English language proficiency. Logistic regression revealed a significant association between examination results and self-satisfaction with English language proficiency (P = 0.048). A positive coefficient (1.802) indicated that passing the examination was more likely with high self-satisfaction for English language proficiency. The above was further confirmed by an odds ratio of 6.063 (95% CI 1.014 to 36.249) for high self-satisfaction for English language proficiency.

Logistic regression revealed no significant association between examination results (passed and referred) and the following factors of interest: revision of lecture handouts before the beginning of the study leave, using textbooks, peer-revision notes and online resources, having an effective study group, self-practice of past paper questions, discussing past paper questions in a study group, sense of interference to academic work due to stress and psycho-social issues, duration of uninterrupted sleep and self-satisfaction with time management, physical fitness, leisure activity, family support, financial support and accommodation (Table 1).

Forty-four per cent (08/18) of undergraduates who occasionally used peer-revision notes had a class at the examination, in comparison to 22% (21/94) of those who frequently used peer-revision notes. Logistic regression revealed a significant association between obtaining class at the examination and using peer-revision notes (P = 0.019). A negative coefficient (− 1.480) indicated that obtaining of a class was less likely with the frequent use of peer-revision notes. The above was further confirmed by an odds ratio of 0.228 (95% CI 0.066 to 0.790) for frequent use of peer-revision notes. Logistic regression revealed no significant association between obtaining a class at the examination and the rest of the factors of interest considered in this study (Table 2).

### Discussion

Examination results were significantly associated with high self-satisfaction for English language proficiency. Previous evidence suggests that a change in medium of learning was associated with academic related stress [25]. Majority of the Sri Lankan medical undergraduates face a change in medium of learning when they enter the university after completion of their schooling. Medical teaching should promote English language proficiency which would help improve the performance of medical undergraduates at examinations.

Using peer-revision notes is popular learning methods among medical undergraduates. However, obtaining of a class was less likely with the frequent use of peer-revision notes. The above finding highlights the need for strict scrutiny of these peer-revision notes. Nevertheless, the use of textbooks and online resources revealed no significant association with the examination results. Also, interference by psycho-social issues had no significant association with academic performance. Inappropriate learning methods and psycho-social issues were shown to be contributive factors for academic stress and subsequent academic failure in prior studies [23, 24]. In contrary to previous literature [16], the duration of uninterrupted sleep also failed to reveal any association with academic performance. It is also surprising to note that having an effective study group and practising of past papers did not influence the examination results.

## Limitations

Findings of a cross-sectional study conducted amongst a single batch of undergraduates of a particular medical school cannot establish a causal association and neither it could be generalised. A national survey at all other medical schools of Sri Lanka would be ideal. However, present data are unique as it was from a medical school located in a rural region, which was the latest to be established in the country. The data on variables of interest were collected following the release of the examination results. However, it would have been ideal if it were collected before the release of the results and subsequently tallied with the examination results. Nevertheless, this is methodologically challenging especially with de-identification and could have led to a high proportion of drop-outs.

## Additional file

**Additional file 1.** Factors associated with the end of pre-clinical stream examination results of medical undergraduates.
